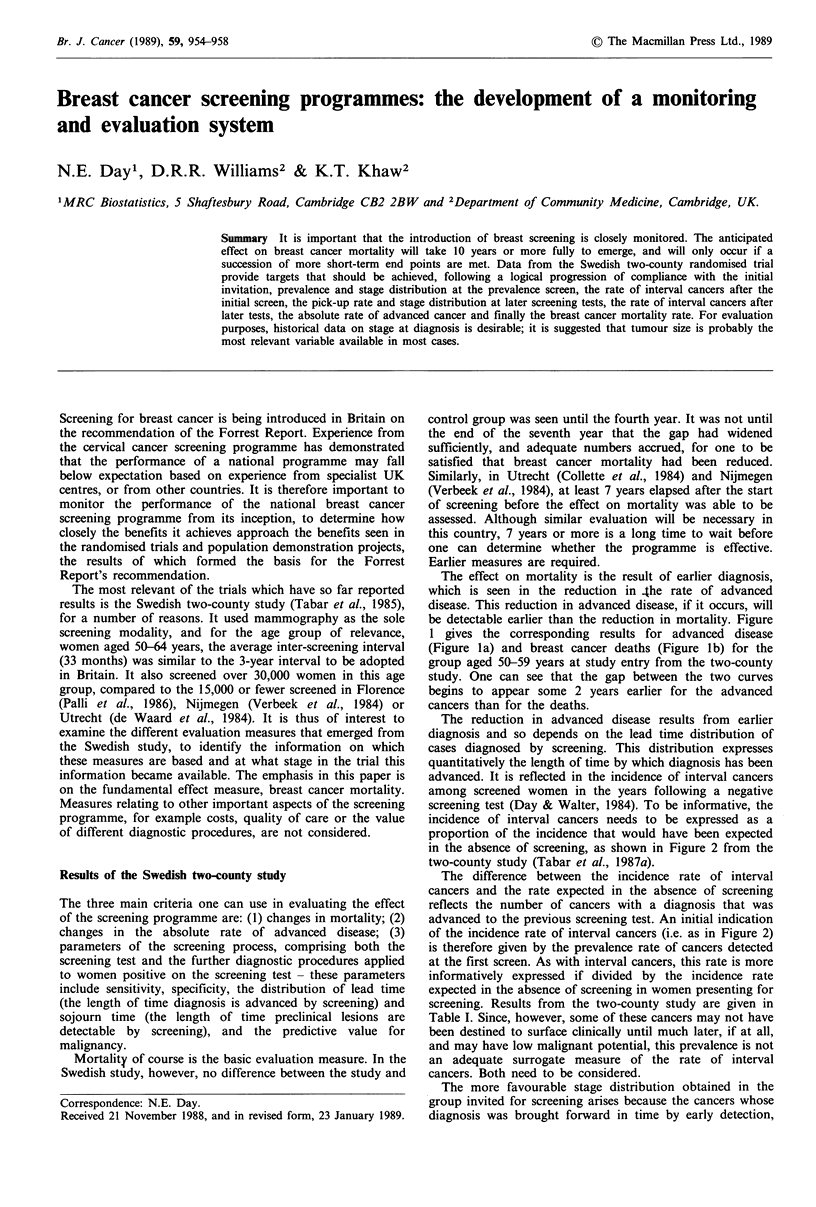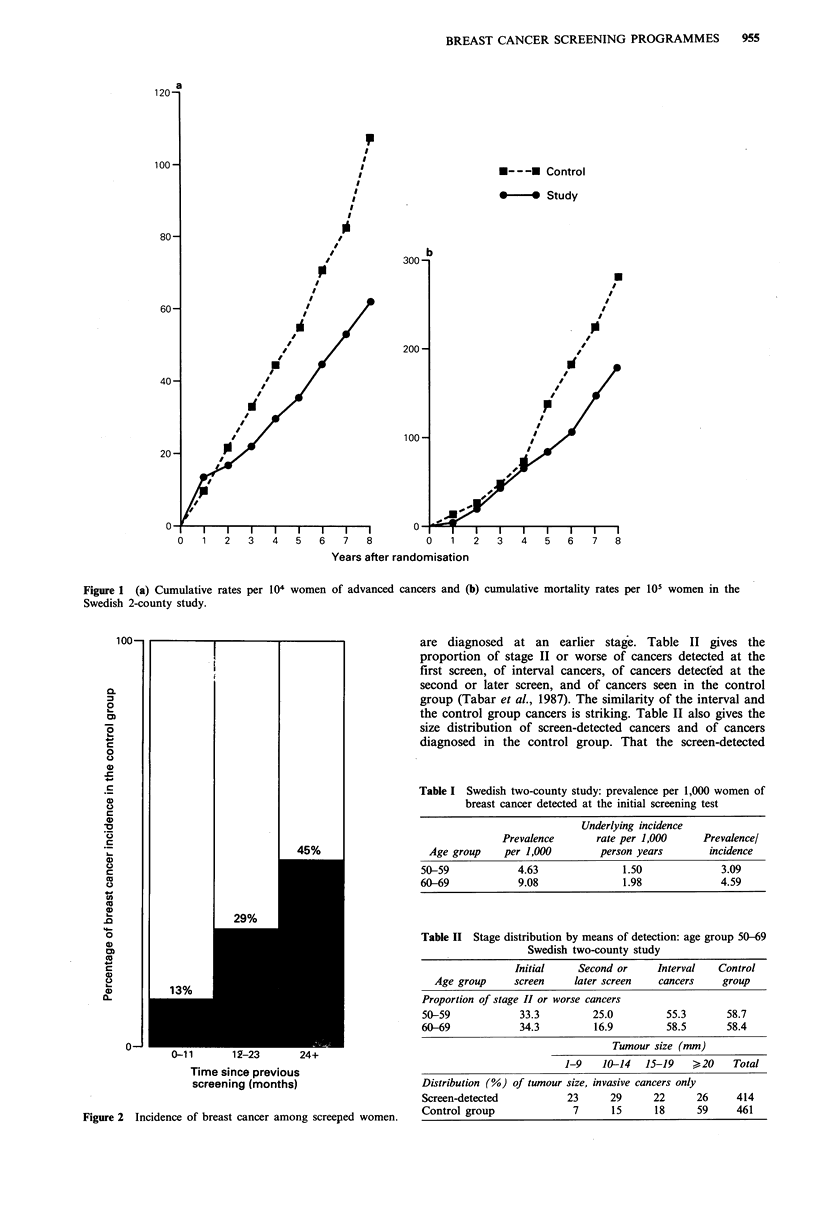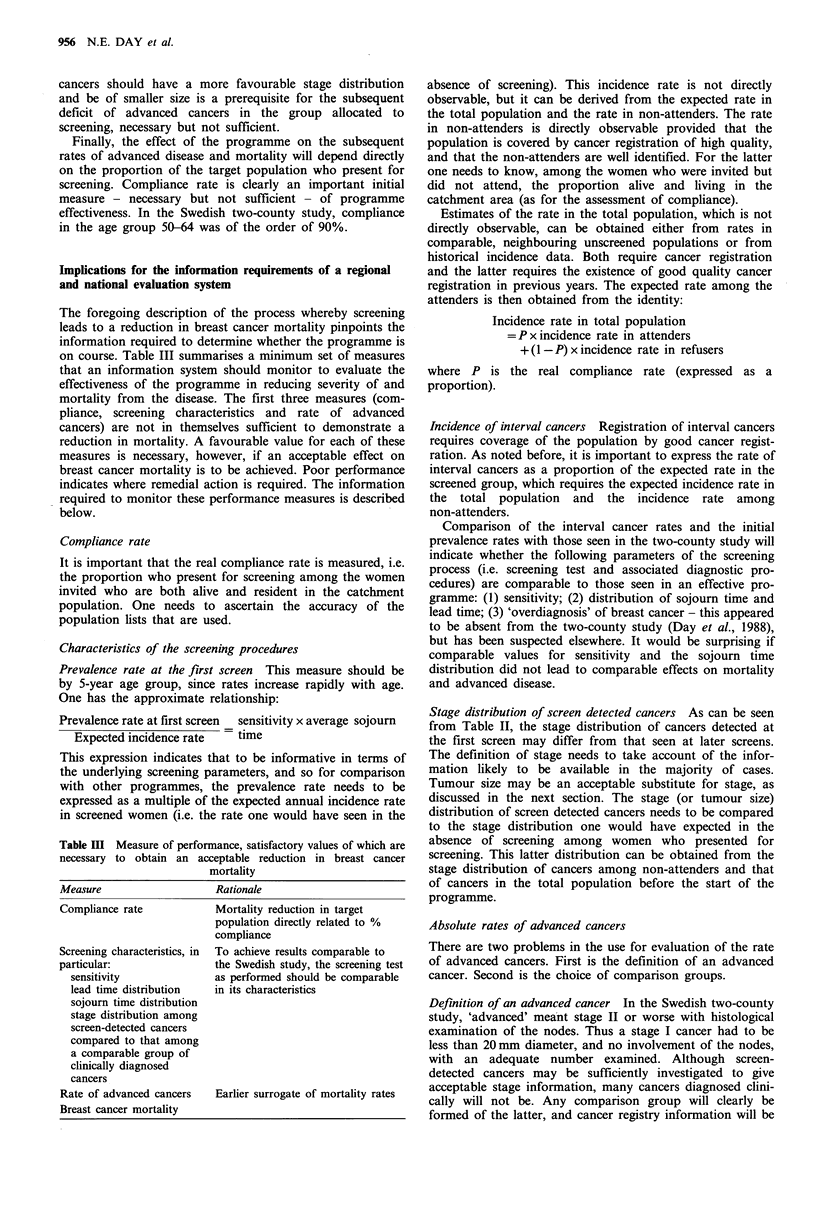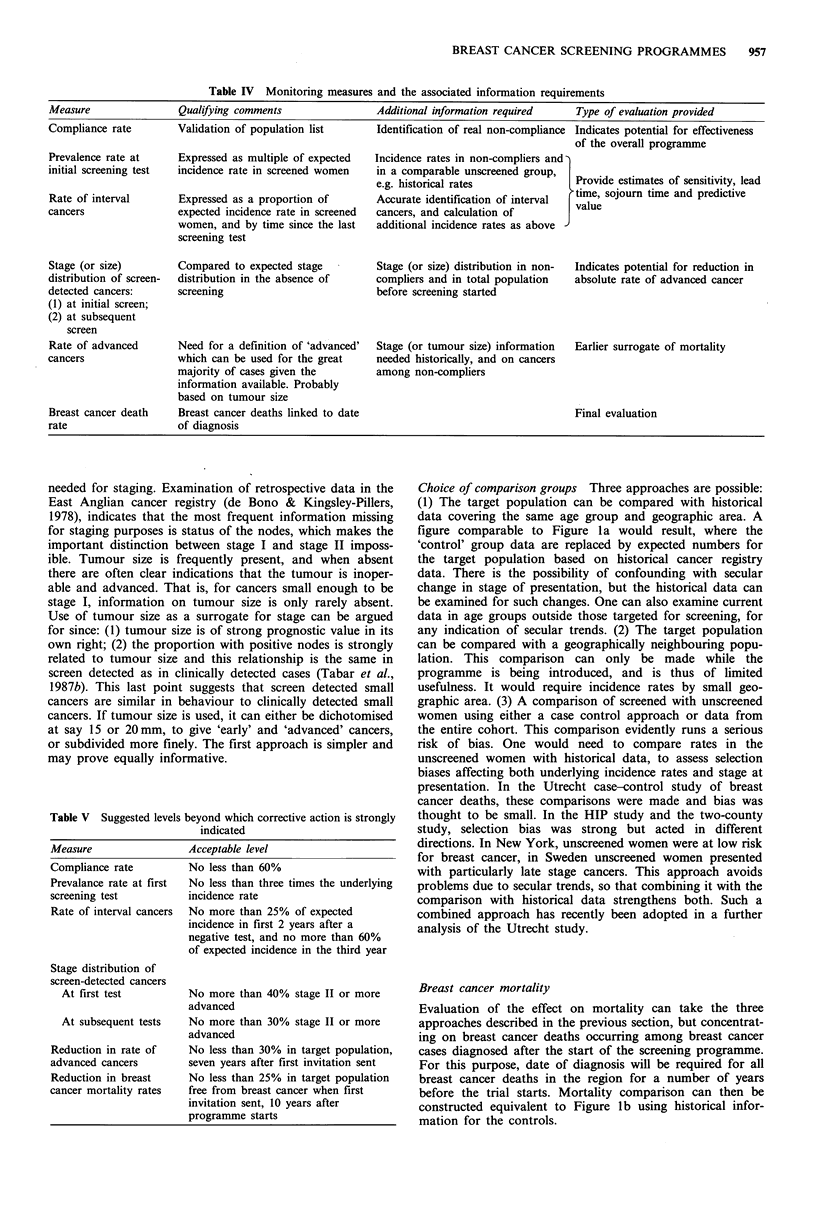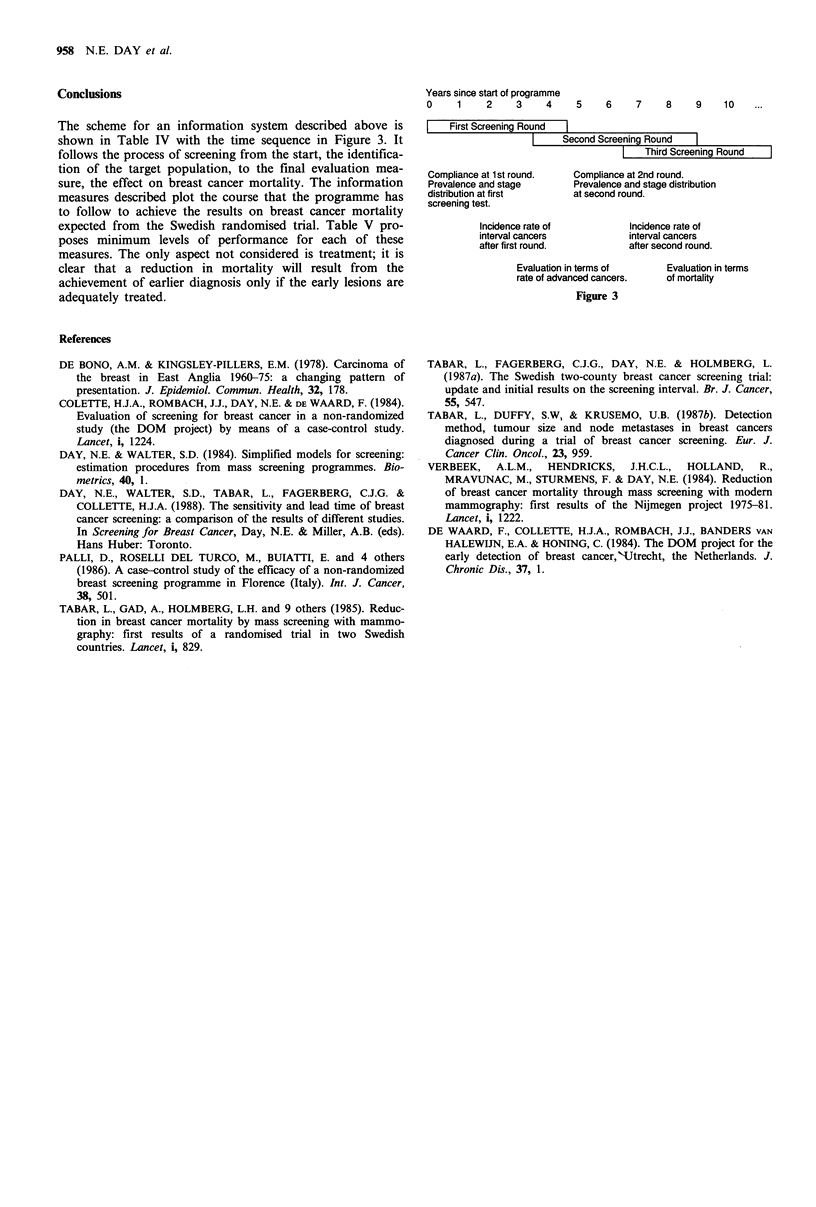# Breast cancer screening programmes: the development of a monitoring and evaluation system.

**DOI:** 10.1038/bjc.1989.203

**Published:** 1989-06

**Authors:** N. E. Day, D. R. Williams, K. T. Khaw

**Affiliations:** MRC Biostatistics, Cambridge, UK.

## Abstract

It is important that the introduction of breast screening is closely monitored. The anticipated effect on breast cancer mortality will take 10 years or more fully to emerge, and will only occur if a succession of more short-term end points are met. Data from the Swedish two-county randomised trial provide targets that should be achieved, following a logical progression of compliance with the initial invitation, prevalence and stage distribution at the prevalence screen, the rate of interval cancers after the initial screen, the pick-up rate and stage distribution at later screening tests, the rate of interval cancers after later tests, the absolute rate of advanced cancer and finally the breast cancer mortality rate. For evaluation purposes, historical data on stage at diagnosis is desirable; it is suggested that tumour size is probably the most relevant variable available in most cases.


*Br. J. Cancer* (1989), **59**, 954–958 © The Macmillan Press Ltd., 1989

### Breast cancer screening programmes: the development of a monitoring and evaluation system

N.E. Day[1], D.R.R. Williams[2] & K.T. Khaw[2]

[1]*MRC Biostatistics, 5 Shaftesbury Road, Cambridge CB2 2BW and* [2]*Department of Community Medicine, Cambridge, UK.*

**Summary** It is important that the introduction of breast screening is closely monitored. The anticipated effect on breast cancer mortality will take 10 years or more fully to emerge, and will only occur if a succession of more short-term end points are met. Data from the Swedish two-county randomised trial provide targets that should be achieved, following a logical progression of compliance with the initial invitation, prevalence and stage distribution at the prevalence screen, the rate of interval cancers after the initial screen, the pick-up rate and stage distribution at later screening tests, the rate of interval cancers after later tests, the absolute rate of advanced cancer and finally the breast cancer mortality rate. For evaluation purposes, historical data on stage at diagnosis is desirable; it is suggested that tumour size is probably the most relevant variable available in most cases.

Screening for breast cancer is being introduced in Britain on the recommendation of the Forrest Report. Experience from the cervical cancer screening programme has demonstrated that the performance of a national programme may fall below expectation based on experience from specialist UK centres, or from other countries. It is therefore important to monitor the performance of the national breast cancer screening programme from its inception, to determine how closely the benefits it achieves approach the benefits seen in the randomised trials and population demonstration projects, the results of which formed the basis for the Forrest Report's recommendation.

The most relevant of the trials which have so far reported results is the Swedish two-county study (Tabar *et al.*, 1985), for a number of reasons. It used mammography as the sole screening modality, and for the age group of relevance, women aged 50–64 years, the average inter-screening interval (33 months) was similar to the 3-year interval to be adopted in Britain. It also screened over 30,000 women in this age group, compared to the 15,000 or fewer screened in Florence (Palli *et al.*, 1986), Nijmegen (Verbeek *et al.*, 1984) or Utrecht (de Waard *et al.*, 1984). It is thus of interest to examine the different evaluation measures that emerged from the Swedish study, to identify the information on which these measures are based and at what stage in the trial this information became available. The emphasis in this paper is on the fundamental effect measure, breast cancer mortality. Measures relating to other important aspects of the screening programme, for example costs, quality of care or the value of different diagnostic procedures, are not considered.

### Results of the Swedish two-county study

The three main criteria one can use in evaluating the effect of the screening programme are: (1) changes in mortality; (2) changes in the absolute rate of advanced disease; (3) parameters of the screening process, comprising both the screening test and the further diagnostic procedures applied to women positive on the screening test – these parameters include sensitivity, specificity, the distribution of lead time (the length of time diagnosis is advanced by screening) and sojourn time (the length of time preclinical lesions are detectable by screening), and the predictive value for malignancy.

Mortality of course is the basic evaluation measure. In the Swedish study, however, no difference between the study and control group was seen until the fourth year. It was not until the end of the seventh year that the gap had widened sufficiently, and adequate numbers accrued, for one to be satisfied that breast cancer mortality had been reduced. Similarly, in Utrecht (Collette *et al.*, 1984) and Nijmegen (Verbeek *et al.*, 1984), at least 7 years elapsed after the start of screening before the effect on mortality was able to be assessed. Although similar evaluation will be necessary in this country, 7 years or more is a long time to wait before one can determine whether the programme is effective. Earlier measures are required.

The effect on mortality is the result of earlier diagnosis, which is seen in the reduction in the rate of advanced disease. This reduction in advanced disease, if it occurs, will be detectable earlier than the reduction in mortality. Figure 1 gives the corresponding results for advanced disease (Figure 1a) and breast cancer deaths (Figure 1b) for the group aged 50–59 years at study entry from the two-county study. One can see that the gap between the two curves begins to appear some 2 years earlier for the advanced cancers than for the deaths.

The reduction in advanced disease results from earlier diagnosis and so depends on the lead time distribution of cases diagnosed by screening. This distribution expresses quantitatively the length of time by which diagnosis has been advanced. It is reflected in the incidence of interval cancers among screened women in the years following a negative screening test (Day & Walter, 1984). To be informative, the incidence of interval cancers needs to be expressed as a proportion of the incidence that would have been expected in the absence of screening, as shown in Figure 2 from the two-county study (Tabar *et al.*, 1987*a*).

The difference between the incidence rate of interval cancers and the rate expected in the absence of screening reflects the number of cancers with a diagnosis that was advanced to the previous screening test. An initial indication of the incidence rate of interval cancers (i.e. as in Figure 2) is therefore given by the prevalence rate of cancers detected at the first screen. As with interval cancers, this rate is more informatively expressed if divided by the incidence rate expected in the absence of screening in women presenting for screening. Results from the two-county study are given in Table I. Since, however, some of these cancers may not have been destined to surface clinically until much later, if at all, and may have low malignant potential, this prevalence is not an adequate surrogate measure of the rate of interval cancers. Both need to be considered.

The more favourable stage distribution obtained in the group invited for screening arises because the cancers whose diagnosis was brought forward in time by early detection,

Correspondence: N.E. Day.
Received 21 November 1988, and in revised form, 23 January 1989.

**Figure 1** (a) Cumulative rates per $10^4$ women of advanced cancers and (b) cumulative mortality rates per $10^5$ women in the Swedish 2-county study.

**Figure 2** Incidence of breast cancer among screened women.

are diagnosed at an earlier stage. Table II gives the proportion of stage II or worse of cancers detected at the first screen, of interval cancers, of cancers detected at the second or later screen, and of cancers seen in the control group (Tabar *et al.*, 1987). The similarity of the interval and the control group cancers is striking. Table II also gives the size distribution of screen-detected cancers and of cancers diagnosed in the control group. That the screen-detected

**Table I** Swedish two-county study: prevalence per 1,000 women of breast cancer detected at the initial screening test

| *Age group* | *Prevalence per 1,000* | *Underlying incidence rate per 1,000 person years* | *Prevalence/ incidence* |
|---|---|---|---|
| 50–59 | 4.63 | 1.50 | 3.09 |
| 60–69 | 9.08 | 1.98 | 4.59 |

**Table II** Stage distribution by means of detection: age group 50–69 Swedish two-county study

| *Age group* | *Initial screen* | *Second or later screen* | *Interval cancers* | *Control group* |
|---|---|---|---|---|
| *Proportion of stage II or worse cancers* | | | | |
| 50–59 | 33.3 | 25.0 | 55.3 | 58.7 |
| 60–69 | 34.3 | 16.9 | 58.5 | 58.4 |

| | *Tumour size (mm)* | | | | |
|---|---|---|---|---|---|
| | *1–9* | *10–14* | *15–19* | *≥20* | *Total* |
| *Distribution (%) of tumour size, invasive cancers only* | | | | | |
| Screen-detected | 23 | 29 | 22 | 26 | 414 |
| Control group | 7 | 15 | 18 | 59 | 461 |

cancers should have a more favourable stage distribution and be of smaller size is a prerequisite for the subsequent deficit of advanced cancers in the group allocated to screening, necessary but not sufficient.

Finally, the effect of the programme on the subsequent rates of advanced disease and mortality will depend directly on the proportion of the target population who present for screening. Compliance rate is clearly an important initial measure – necessary but not sufficient – of programme effectiveness. In the Swedish two-county study, compliance in the age group 50–64 was of the order of 90%.

**Implications for the information requirements of a regional and national evaluation system**

The foregoing description of the process whereby screening leads to a reduction in breast cancer mortality pinpoints the information required to determine whether the programme is on course. Table III summarises a minimum set of measures that an information system should monitor to evaluate the effectiveness of the programme in reducing severity of and mortality from the disease. The first three measures (compliance, screening characteristics and rate of advanced cancers) are not in themselves sufficient to demonstrate a reduction in mortality. A favourable value for each of these measures is necessary, however, if an acceptable effect on breast cancer mortality is to be achieved. Poor performance indicates where remedial action is required. The information required to monitor these performance measures is described below.

*Compliance rate*

It is important that the real compliance rate is measured, i.e. the proportion who present for screening among the women invited who are both alive and resident in the catchment population. One needs to ascertain the accuracy of the population lists that are used.

*Characteristics of the screening procedures*

*Prevalence rate at the first screen* This measure should be by 5-year age group, since rates increase rapidly with age. One has the approximate relationship:

$$\frac{\text{Prevalence rate at first screen}}{\text{Expected incidence rate}} = \text{sensitivity} \times \text{average sojourn time}$$

This expression indicates that to be informative in terms of the underlying screening parameters, and so for comparison with other programmes, the prevalence rate needs to be expressed as a multiple of the expected annual incidence rate in screened women (i.e. the rate one would have seen in the absence of screening). This incidence rate is not directly observable, but it can be derived from the expected rate in the total population and the rate in non-attenders. The rate in non-attenders is directly observable provided that the population is covered by cancer registration of high quality, and that the non-attenders are well identified. For the latter one needs to know, among the women who were invited but did not attend, the proportion alive and living in the catchment area (as for the assessment of compliance).

**Table III** Measure of performance, satisfactory values of which are necessary to obtain an acceptable reduction in breast cancer mortality

| *Measure* | *Rationale* |
|---|---|
| Compliance rate | Mortality reduction in target population directly related to % compliance |
| Screening characteristics, in particular: sensitivity; lead time distribution; sojourn time distribution; stage distribution among screen-detected cancers compared to that among a comparable group of clinically diagnosed cancers | To achieve results comparable to the Swedish study, the screening test as performed should be comparable in its characteristics |
| Rate of advanced cancers | Earlier surrogate of mortality rates |
| Breast cancer mortality | |

Estimates of the rate in the total population, which is not directly observable, can be obtained either from rates in comparable, neighbouring unscreened populations or from historical incidence data. Both require cancer registration and the latter requires the existence of good quality cancer registration in previous years. The expected rate among the attenders is then obtained from the identity:

$$\begin{aligned}&\text{Incidence rate in total population}\\&= P \times \text{incidence rate in attenders}\\&\quad + (1-P) \times \text{incidence rate in refusers}\end{aligned}$$

where $P$ is the real compliance rate (expressed as a proportion).

*Incidence of interval cancers* Registration of interval cancers requires coverage of the population by good cancer registration. As noted before, it is important to express the rate of interval cancers as a proportion of the expected rate in the screened group, which requires the expected incidence rate in the total population and the incidence rate among non-attenders.

Comparison of the interval cancer rates and the initial prevalence rates with those seen in the two-county study will indicate whether the following parameters of the screening process (i.e. screening test and associated diagnostic procedures) are comparable to those seen in an effective programme: (1) sensitivity; (2) distribution of sojourn time and lead time; (3) 'overdiagnosis' of breast cancer – this appeared to be absent from the two-county study (Day *et al.*, 1988), but has been suspected elsewhere. It would be surprising if comparable values for sensitivity and the sojourn time distribution did not lead to comparable effects on mortality and advanced disease.

*Stage distribution of screen detected cancers* As can be seen from Table II, the stage distribution of cancers detected at the first screen may differ from that seen at later screens. The definition of stage needs to take account of the information likely to be available in the majority of cases. Tumour size may be an acceptable substitute for stage, as discussed in the next section. The stage (or tumour size) distribution of screen detected cancers needs to be compared to the stage distribution one would have expected in the absence of screening among women who presented for screening. This latter distribution can be obtained from the stage distribution of cancers among non-attenders and that of cancers in the total population before the start of the programme.

*Absolute rates of advanced cancers*

There are two problems in the use for evaluation of the rate of advanced cancers. First is the definition of an advanced cancer. Second is the choice of comparison groups.

*Definition of an advanced cancer* In the Swedish two-county study, 'advanced' meant stage II or worse with histological examination of the nodes. Thus a stage I cancer had to be less than 20 mm diameter, and no involvement of the nodes, with an adequate number examined. Although screen-detected cancers may be sufficiently investigated to give acceptable stage information, many cancers diagnosed clinically will not be. Any comparison group will clearly be formed of the latter, and cancer registry information will be

**Table IV** Monitoring measures and the associated information requirements

| *Measure* | *Qualifying comments* | *Additional information required* | *Type of evaluation provided* |
|---|---|---|---|
| Compliance rate | Validation of population list | Identification of real non-compliance | Indicates potential for effectiveness of the overall programme |
| Prevalence rate at initial screening test | Expressed as multiple of expected incidence rate in screened women | Incidence rates in non-compliers and in a comparable unscreened group, e.g. historical rates | Provide estimates of sensitivity, lead time, sojourn time and predictive value |
| Rate of interval cancers | Expressed as a proportion of expected incidence rate in screened women, and by time since the last screening test | Accurate identification of interval cancers, and calculation of additional incidence rates as above | |
| Stage (or size) distribution of screen-detected cancers: (1) at initial screen; (2) at subsequent screen | Compared to expected stage distribution in the absence of screening | Stage (or size) distribution in non-compliers and in total population before screening started | Indicates potential for reduction in absolute rate of advanced cancer |
| Rate of advanced cancers | Need for a definition of 'advanced' which can be used for the great majority of cases given the information available. Probably based on tumour size | Stage (or tumour size) information needed historically, and on cancers among non-compliers | Earlier surrogate of mortality |
| Breast cancer death rate | Breast cancer deaths linked to date of diagnosis | | Final evaluation |

needed for staging. Examination of retrospective data in the East Anglian cancer registry (de Bono & Kingsley-Pillers, 1978), indicates that the most frequent information missing for staging purposes is status of the nodes, which makes the important distinction between stage I and stage II impossible. Tumour size is frequently present, and when absent there are often clear indications that the tumour is inoperable and advanced. That is, for cancers small enough to be stage I, information on tumour size is only rarely absent. Use of tumour size as a surrogate for stage can be argued for since: (1) tumour size is of strong prognostic value in its own right; (2) the proportion with positive nodes is strongly related to tumour size and this relationship is the same in screen detected as in clinically detected cases (Tabar *et al.*, 1987*b*). This last point suggests that screen detected small cancers are similar in behaviour to clinically detected small cancers. If tumour size is used, it can either be dichotomised at say 15 or 20 mm, to give 'early' and 'advanced' cancers, or subdivided more finely. The first approach is simpler and may prove equally informative.

**Table V** Suggested levels beyond which corrective action is strongly indicated

| *Measure* | *Acceptable level* |
|---|---|
| Compliance rate | No less than 60% |
| Prevalance rate at first screening test | No less than three times the underlying incidence rate |
| Rate of interval cancers | No more than 25% of expected incidence in first 2 years after a negative test, and no more than 60% of expected incidence in the third year |
| Stage distribution of screen-detected cancers | |
| At first test | No more than 40% stage II or more advanced |
| At subsequent tests | No more than 30% stage II or more advanced |
| Reduction in rate of advanced cancers | No less than 30% in target population, seven years after first invitation sent |
| Reduction in breast cancer mortality rates | No less than 25% in target population free from breast cancer when first invitation sent, 10 years after programme starts |

*Choice of comparison groups* Three approaches are possible: (1) The target population can be compared with historical data covering the same age group and geographic area. A figure comparable to Figure 1a would result, where the 'control' group data are replaced by expected numbers for the target population based on historical cancer registry data. There is the possibility of confounding with secular change in stage of presentation, but the historical data can be examined for such changes. One can also examine current data in age groups outside those targeted for screening, for any indication of secular trends. (2) The target population can be compared with a geographically neighbouring population. This comparison can only be made while the programme is being introduced, and is thus of limited usefulness. It would require incidence rates by small geographic area. (3) A comparison of screened with unscreened women using either a case control approach or data from the entire cohort. This comparison evidently runs a serious risk of bias. One would need to compare rates in the unscreened women with historical data, to assess selection biases affecting both underlying incidence rates and stage at presentation. In the Utrecht case–control study of breast cancer deaths, these comparisons were made and bias was thought to be small. In the HIP study and the two-county study, selection bias was strong but acted in different directions. In New York, unscreened women were at low risk for breast cancer, in Sweden unscreened women presented with particularly late stage cancers. This approach avoids problems due to secular trends, so that combining it with the comparison with historical data strengthens both. Such a combined approach has recently been adopted in a further analysis of the Utrecht study.

### *Breast cancer mortality*

Evaluation of the effect on mortality can take the three approaches described in the previous section, but concentrating on breast cancer deaths occurring among breast cancer cases diagnosed after the start of the screening programme. For this purpose, date of diagnosis will be required for all breast cancer deaths in the region for a number of years before the trial starts. Mortality comparison can then be constructed equivalent to Figure 1b using historical information for the controls.

### Conclusions

The scheme for an information system described above is shown in Table IV with the time sequence in Figure 3. It follows the process of screening from the start, the identification of the target population, to the final evaluation measure, the effect on breast cancer mortality. The information measures described plot the course that the programme has to follow to achieve the results on breast cancer mortality expected from the Swedish randomised trial. Table V proposes minimum levels of performance for each of these measures. The only aspect not considered is treatment; it is clear that a reduction in mortality will result from the achievement of earlier diagnosis only if the early lesions are adequately treated.

**Figure 3**

### References

DE BONO, A.M. & KINGSLEY-PILLERS, E.M. (1978). Carcinoma of the breast in East Anglia 1960–75: a changing pattern of presentation. *J. Epidemiol. Commun. Health*, **32**, 178.

COLETTE, H.J.A., ROMBACH, J.J., DAY, N.E. & DE WAARD, F. (1984). Evaluation of screening for breast cancer in a non-randomized study (the DOM project) by means of a case-control study. *Lancet*, **i**, 1224.

DAY, N.E. & WALTER, S.D. (1984). Simplified models for screening: estimation procedures from mass screening programmes. *Biometrics*, **40**, 1.

DAY, N.E., WALTER, S.D., TABAR, L., FAGERBERG, C.J.G. & COLLETTE, H.J.A. (1988). The sensitivity and lead time of breast cancer screening: a comparison of the results of different studies. In *Screening for Breast Cancer*, Day, N.E. & Miller, A.B. (eds). Hans Huber: Toronto.

PALLI, D., ROSELLI DEL TURCO, M., BUIATTI, E. and 4 others (1986). A case–control study of the efficacy of a non-randomized breast screening programme in Florence (Italy). *Int. J. Cancer*, **38**, 501.

TABAR, L., GAD, A., HOLMBERG, L.H. and 9 others (1985). Reduction in breast cancer mortality by mass screening with mammography: first results of a randomised trial in two Swedish countries. *Lancet*, **i**, 829.

TABAR, L., FAGERBERG, C.J.G., DAY, N.E. & HOLMBERG, L. (1987*a*). The Swedish two-county breast cancer screening trial: update and initial results on the screening interval. *Br. J. Cancer*, **55**, 547.

TABAR, L., DUFFY, S.W, & KRUSEMO, U.B. (1987*b*). Detection method, tumour size and node metastases in breast cancers diagnosed during a trial of breast cancer screening. *Eur. J. Cancer Clin. Oncol.*, **23**, 959.

VERBEEK, A.L.M., HENDRICKS, J.H.C.L., HOLLAND, R., MRAVUNAC, M., STURMENS, F. & DAY, N.E. (1984). Reduction of breast cancer mortality through mass screening with modern mammography: first results of the Nijmegen project 1975–81. *Lancet*, **i**, 1222.

DE WAARD, F., COLLETTE, H.J.A., ROMBACH, J.J., BANDERS VAN HALEWIJN, E.A. & HONING, C. (1984). The DOM project for the early detection of breast cancer, Utrecht, the Netherlands. *J. Chronic Dis.*, **37**, 1.